# Supplementary material for: Bayesian inference of ancestral dates on bacterial phylogenetic trees
Source: Nucleic Acids Res. 2018 Sep 3;46(22):e134. doi: 10.1093/nar/gky783 (PMC6294524; doi:10.1093/nar/gky783)
Supplement: Supplementary Data [file gky783_supplemental_files.pdf]

Supplementary material for “Bayesian inference of ancestral dates on bacterial phylogenetic trees”

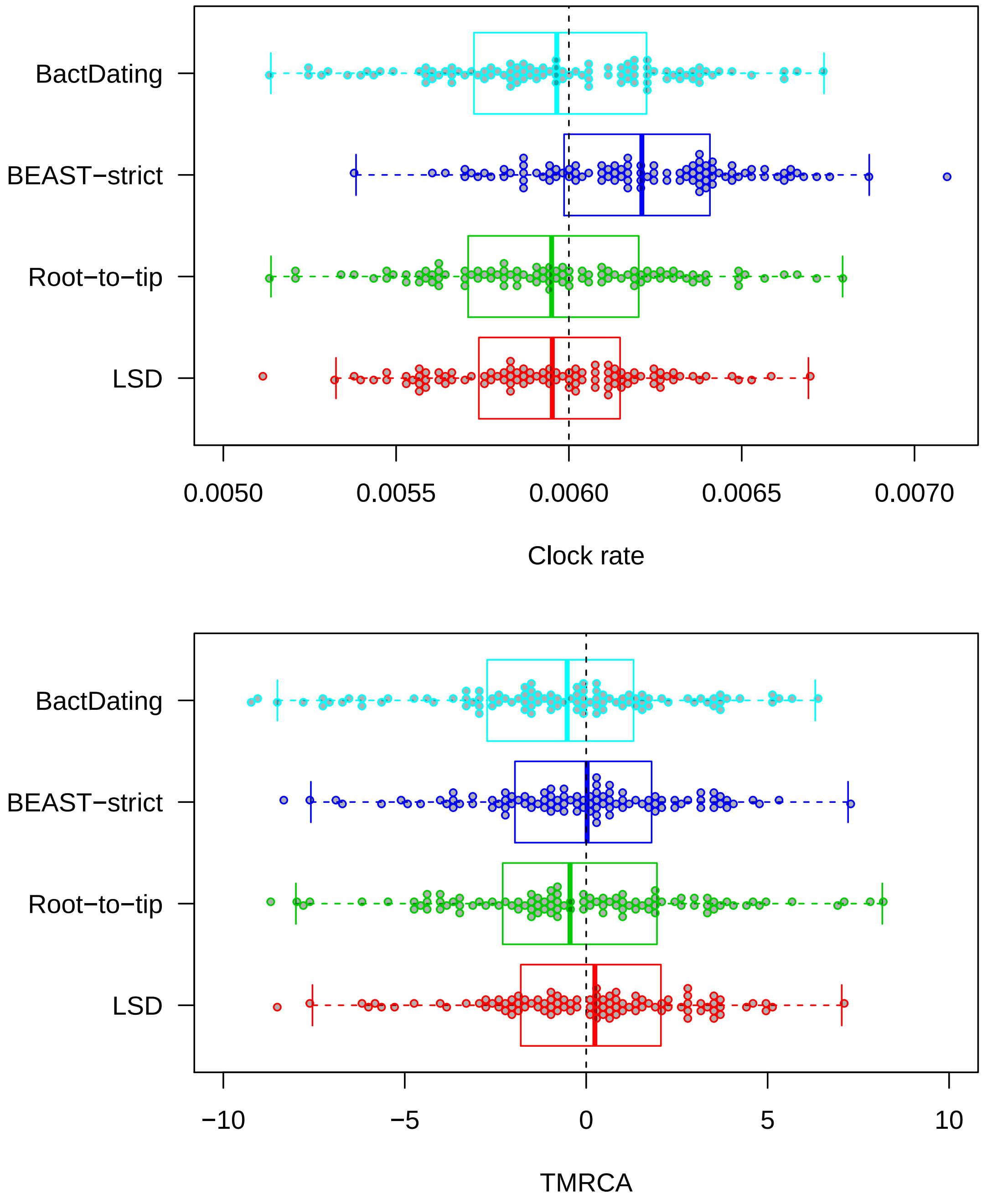

Figure S1. Comparison of BactDating and other methods for the inference of the clock rate (top) and TMRCA (bottom) on the dataset from To et al (2016) 750-3-25 generated with a strict clock.

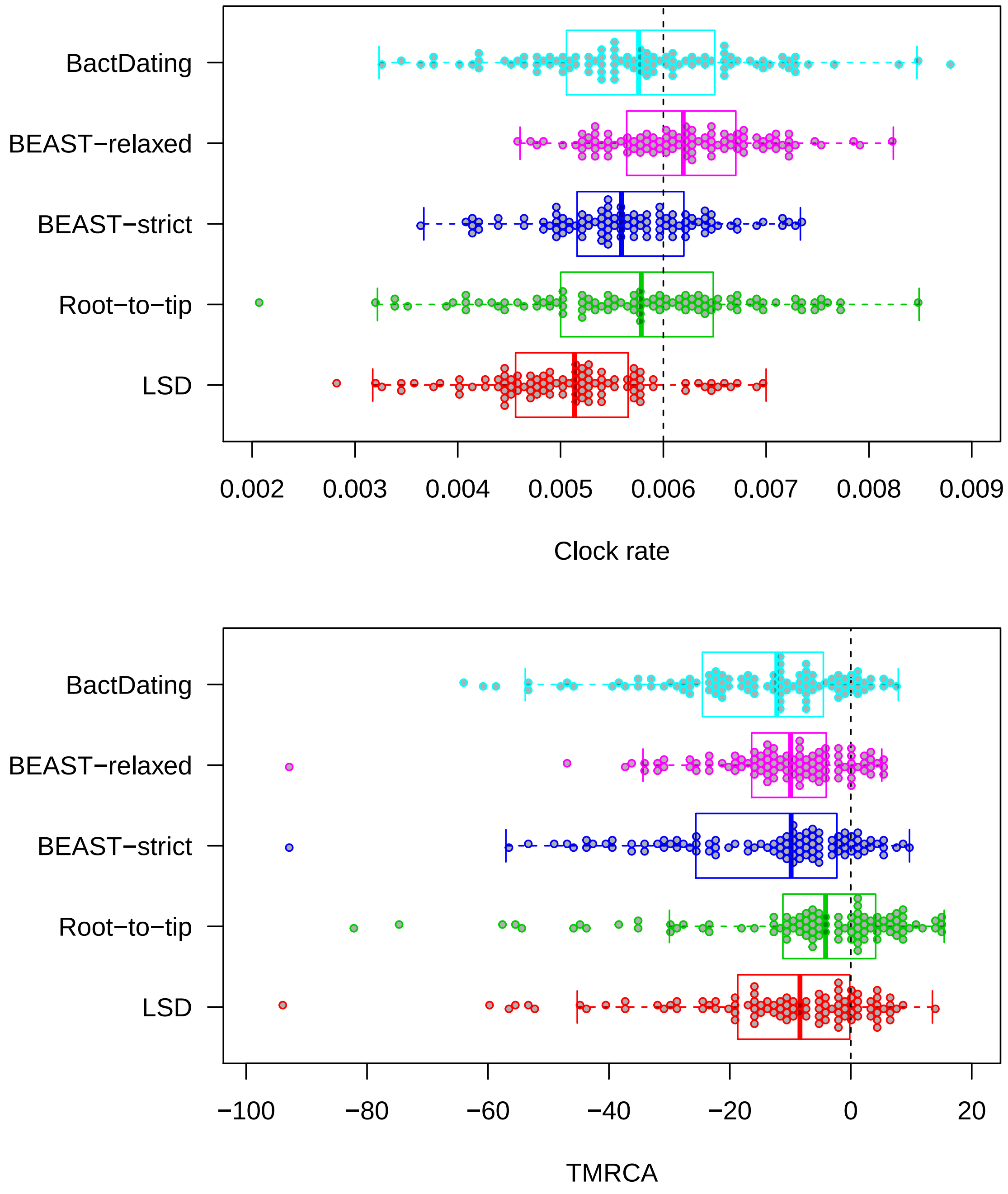

Figure S2. Comparison of BactDating and other methods for the inference of the clock rate (top) and TMRCA (bottom) on the dataset from To et al (2016) 750-3-25 generated with a relaxed clock.

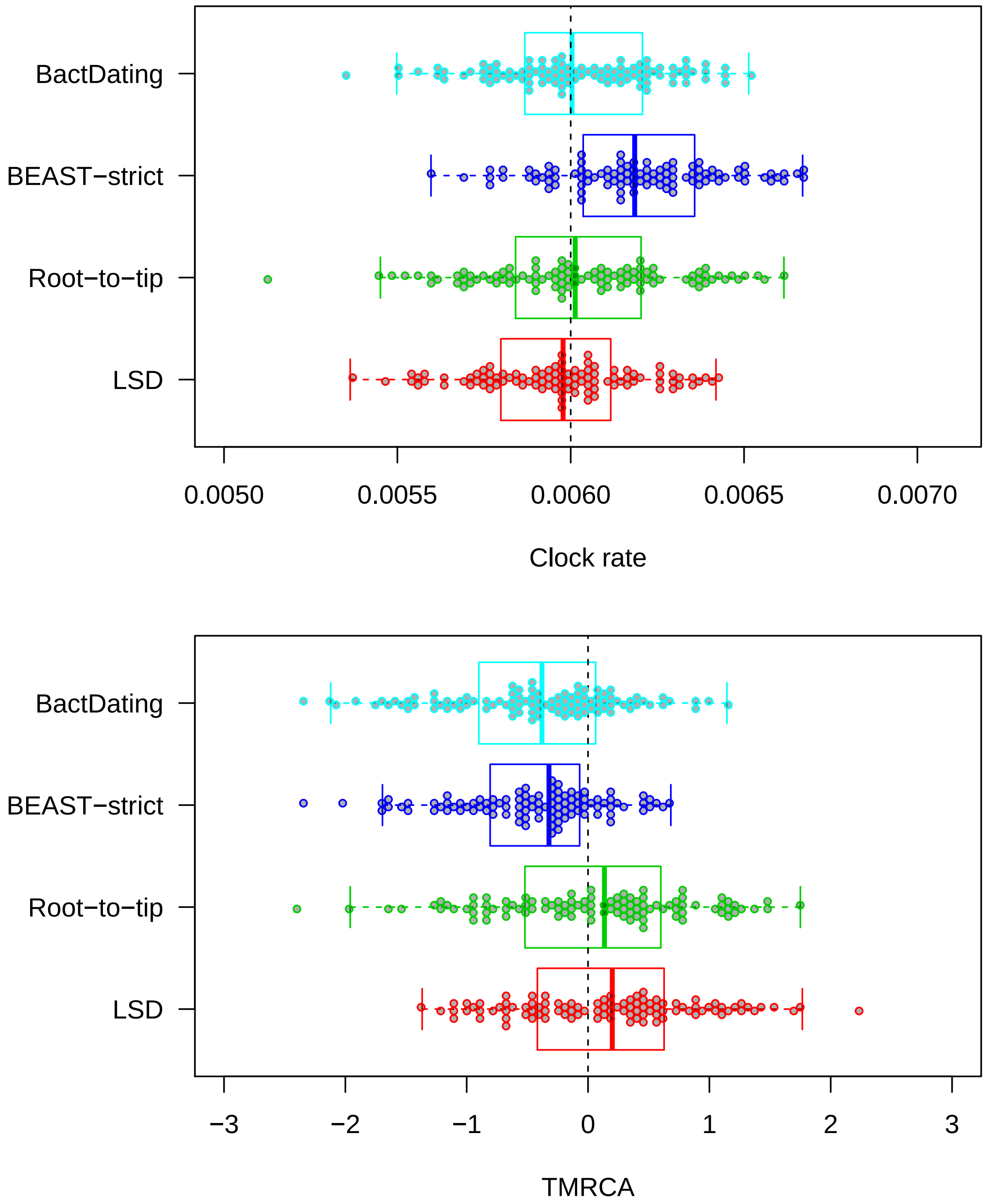

Figure S3. Comparison of BactDating and other methods for the inference of the clock rate (top) and TMRCA (bottom) on the dataset from To et al (2016) 750-11-10 generated with a strict clock.

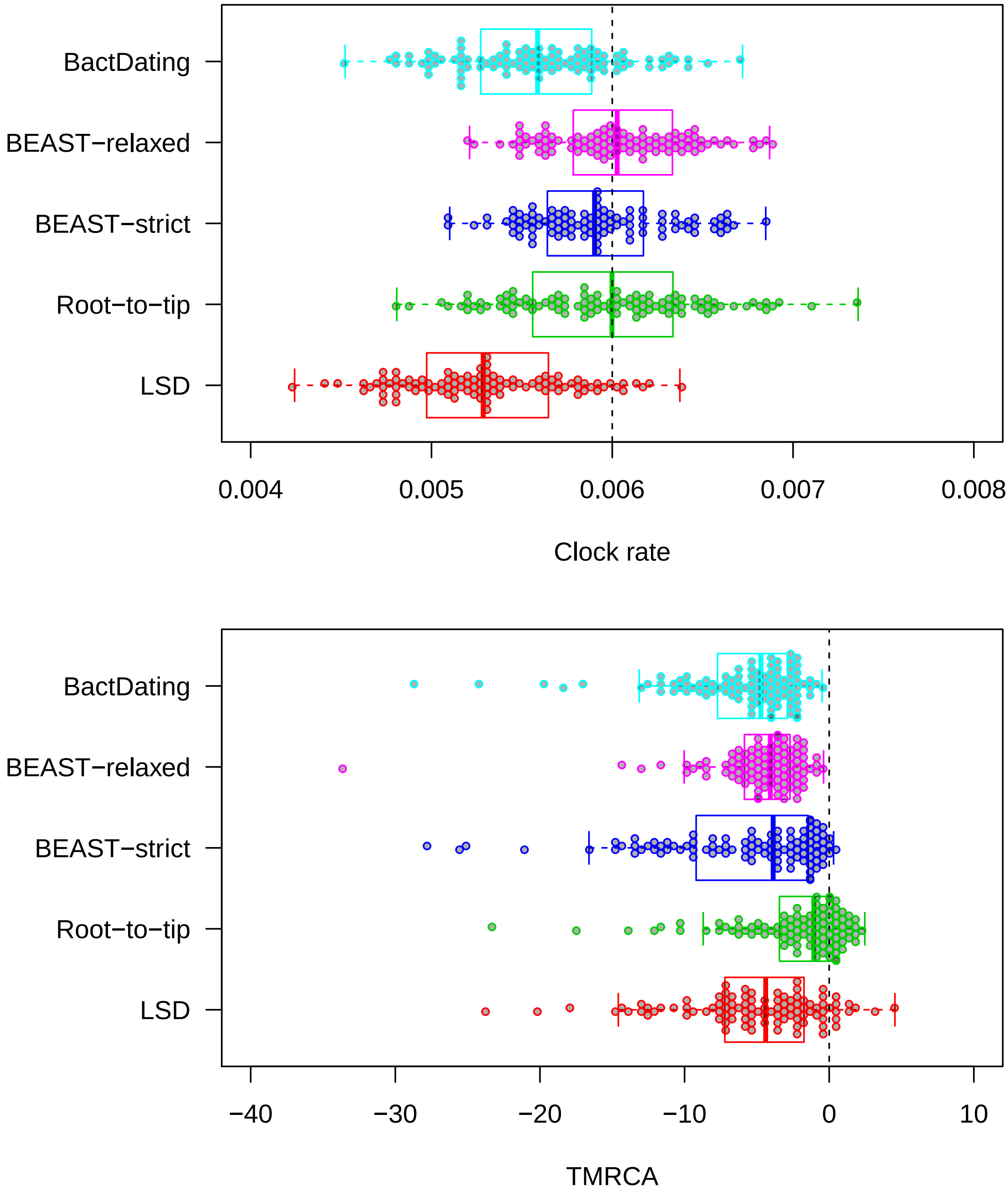

Figure S4. Comparison of BactDating and other methods for the inference of the clock rate (top) and TMRCA (bottom) on the dataset from To et al (2016) 750-11-10 generated with a relaxed clock.

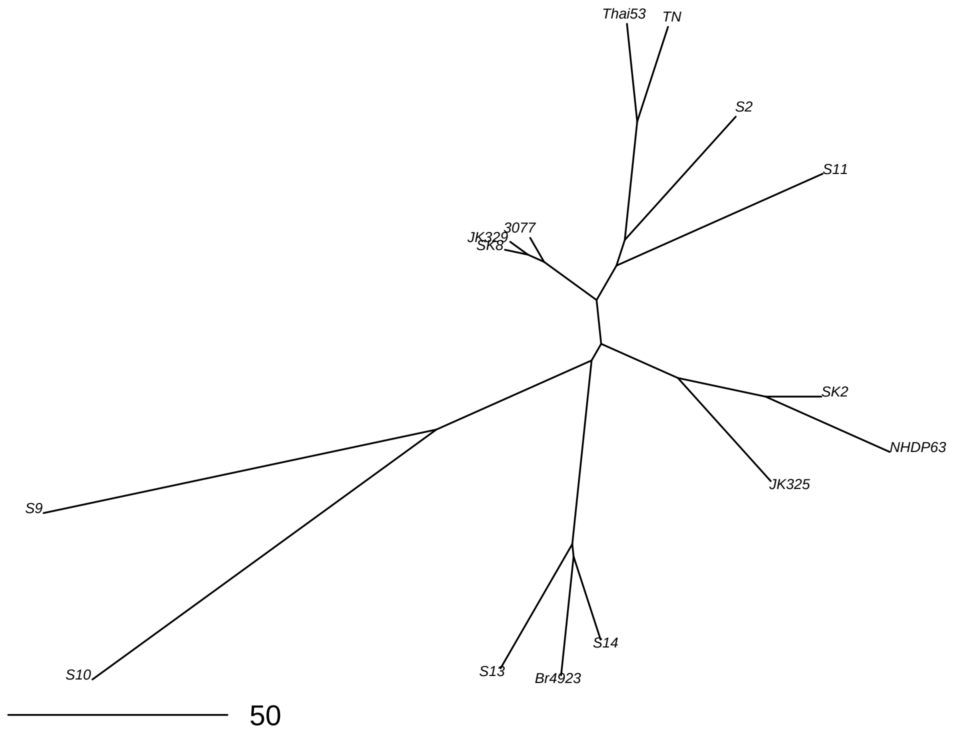

Figure S5. Phylogenetic tree of the *Mycobacterium leprae* dataset. This tree was built using PhyML version 3.1 under a GTR+Γ substitution model with a Γ rate heterogeneity with four classes (options `-m GTR -b 0 -c 4 -s SPR`).

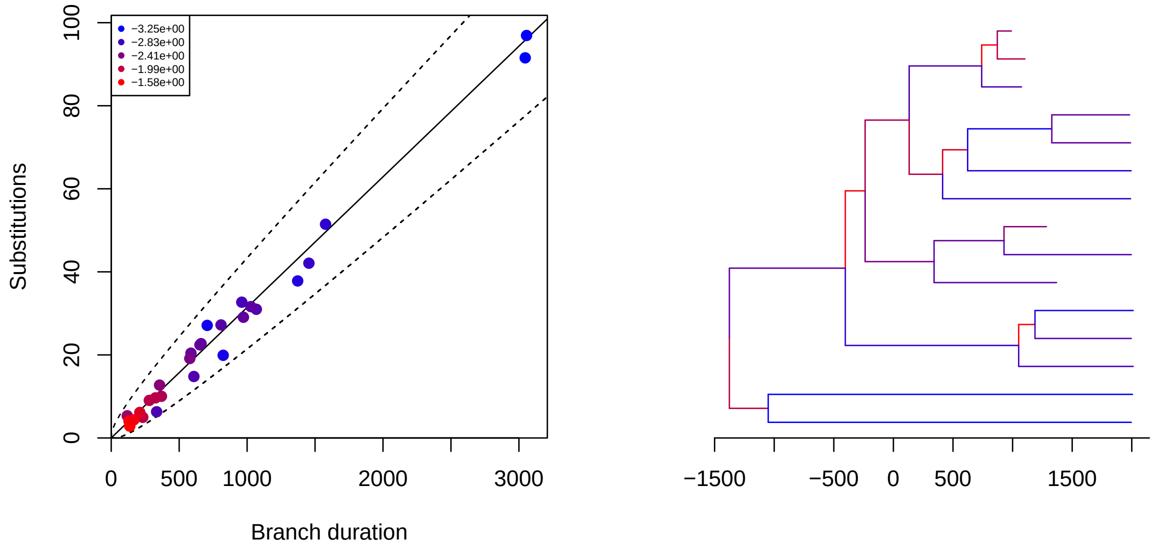

Figure S6. Likelihood contribution of each branch in the *Mycobacterium leprae* timed tree.

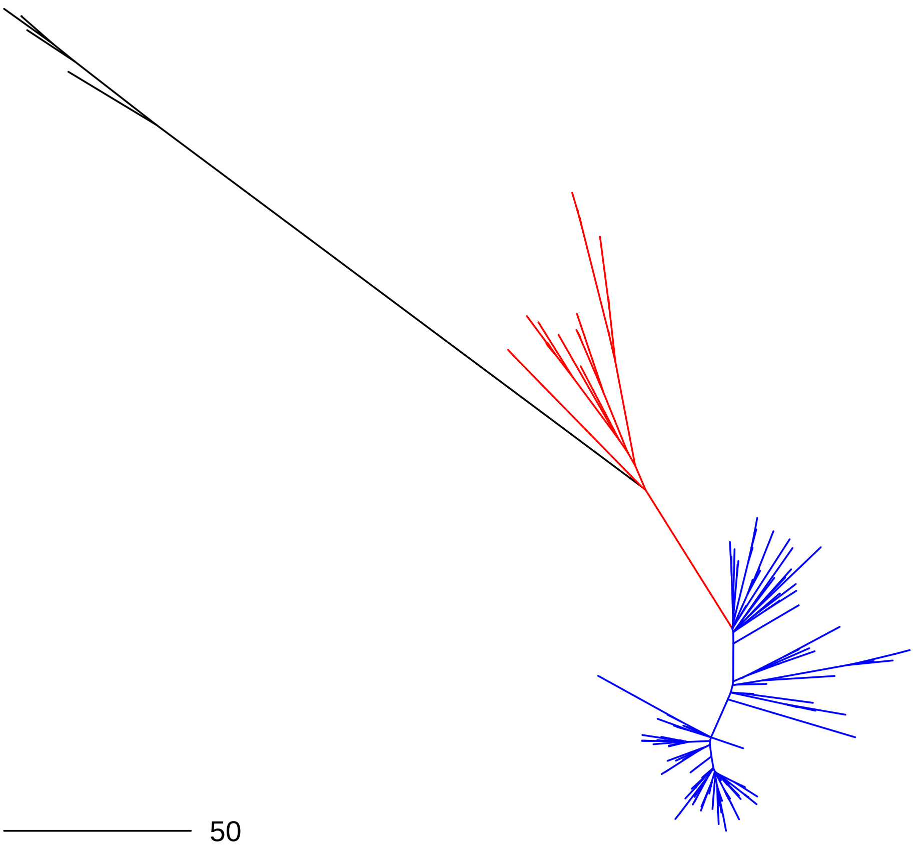

Figure S7. Phylogenetic tree of the *Shigella sonnei* VN dataset. The group colored in black represents outgroups used to root the tree and therefore excluded in the phylogenetic analysis. The group colored in blue represents the VN clone. This tree was built using PhyML version 3.1 under a GTR+Γ substitution model with a Γ rate heterogeneity with four classes (options `-m GTR -b 0 -c 4 -s SPR`).

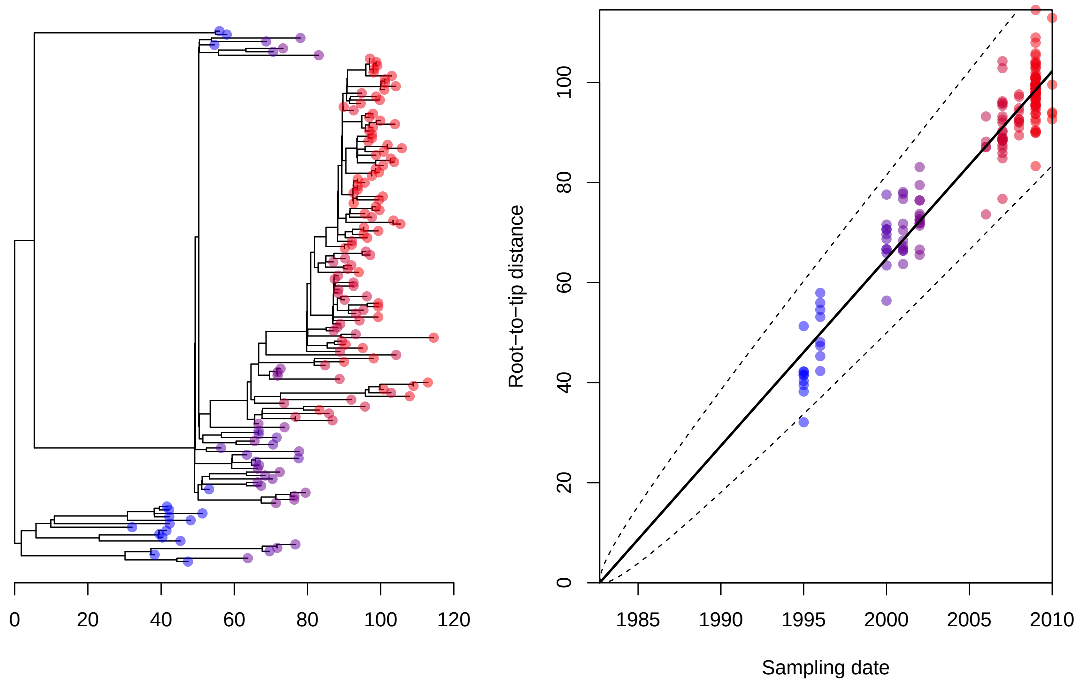

Figure S8. Linear regression analysis of root-to-tip distances versus isolation dates for the *Shigella sonnei* VN dataset.

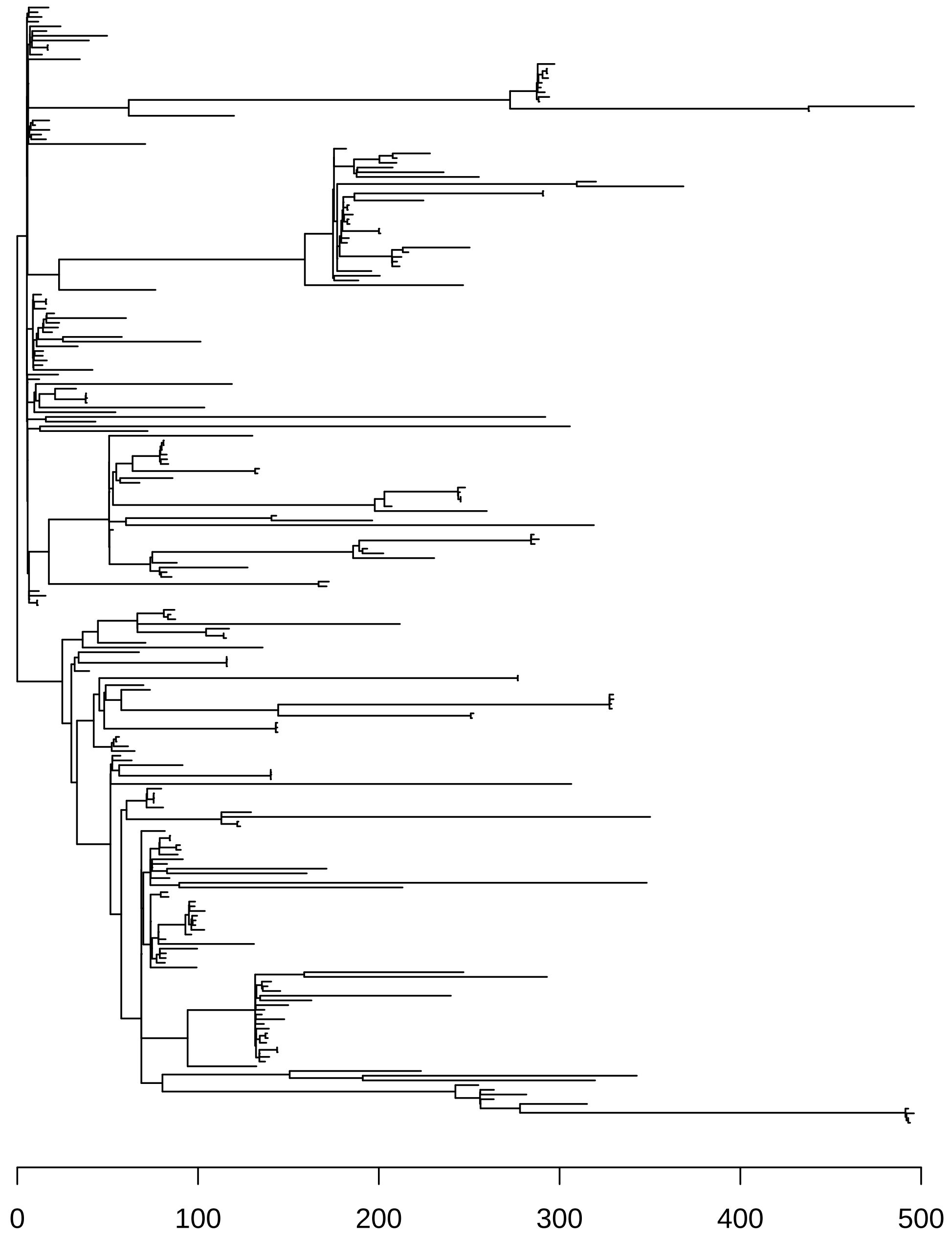

Figure S9. Phylogenetic tree of the *Streptococcus pneumoniae* PMEN1 dataset obtained using RAxML. This tree was built using RAxML version 8.2.8 under a GTR+Γ substitution model (options `-f d -p 1 -m GTRGAMMA`).

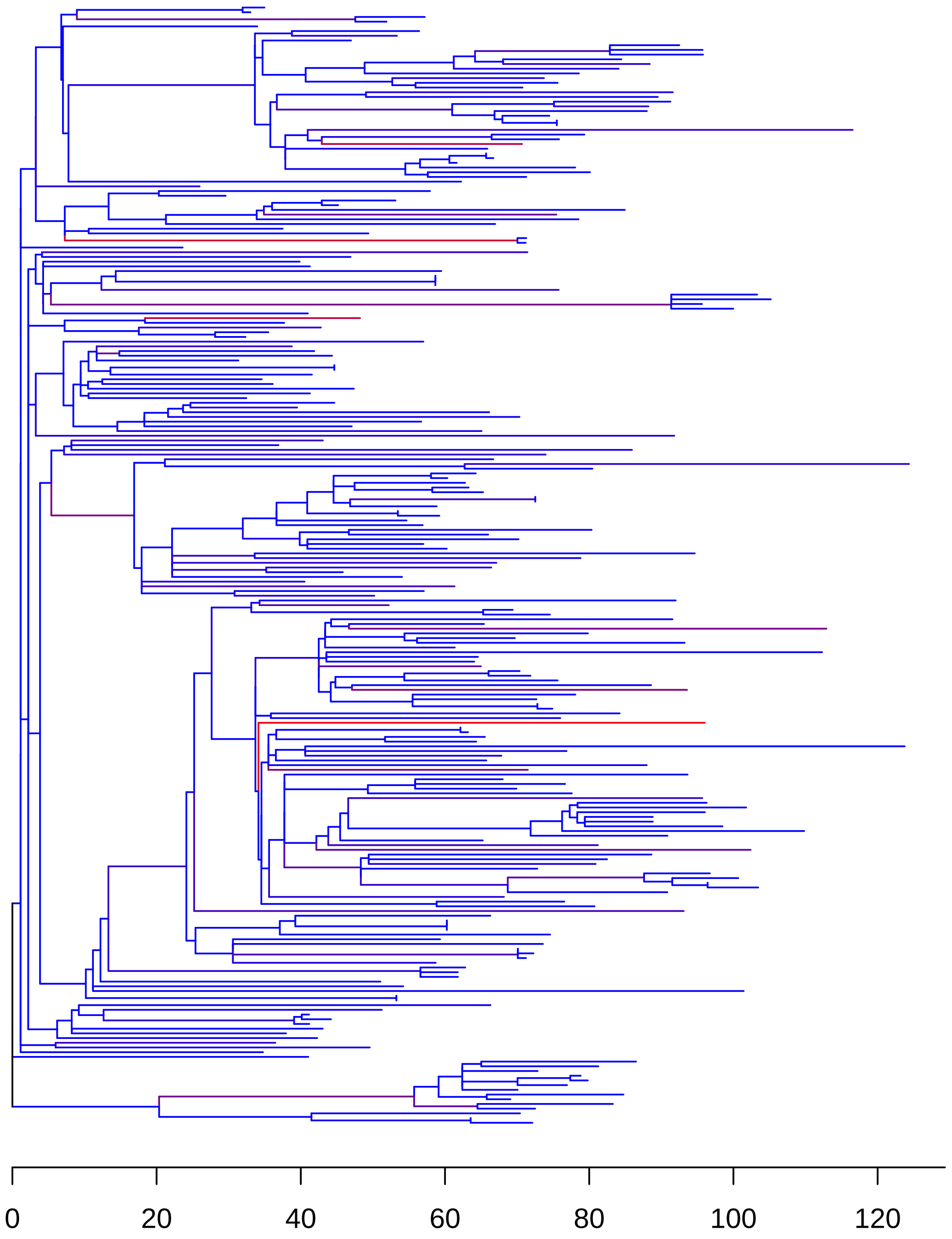

Figure S10. Phylogenetic tree of the *Streptococcus pneumoniae* PMEN1 dataset obtained using Gubbins, with branches coloured from blue to red proportionally with the length of recombined material.

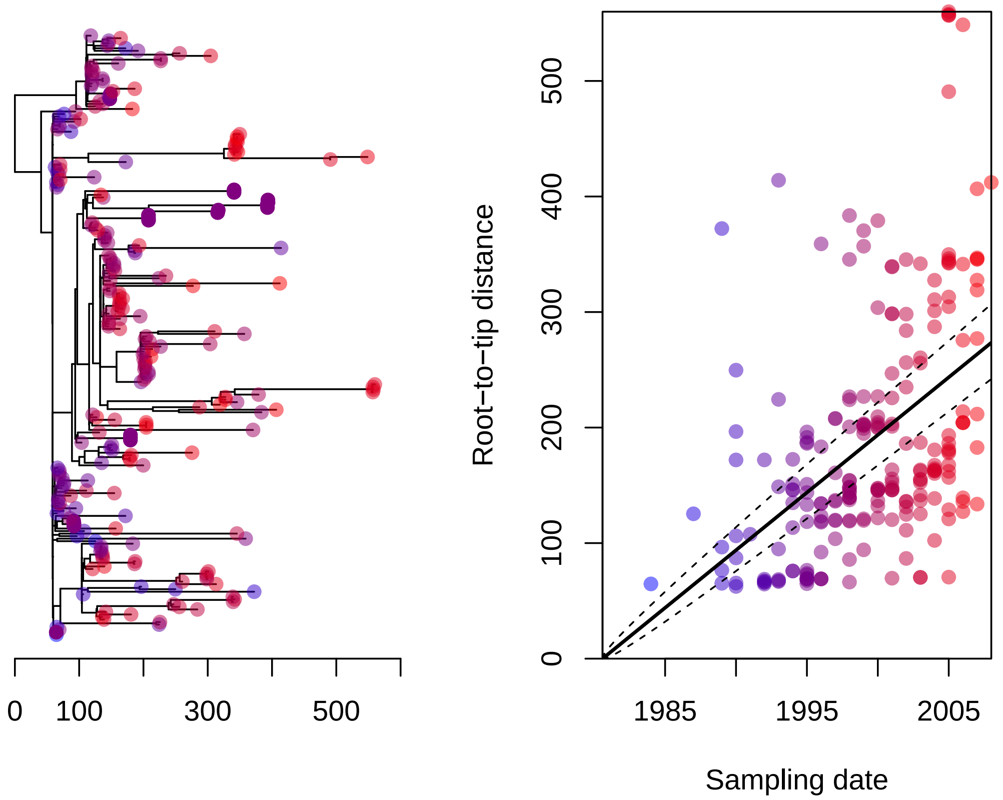

Figure S11. Linear regression analysis of root-to-tip distances versus isolation dates, based on the RAxML tree of the *Streptococcus pneumoniae* PMEN1 dataset.

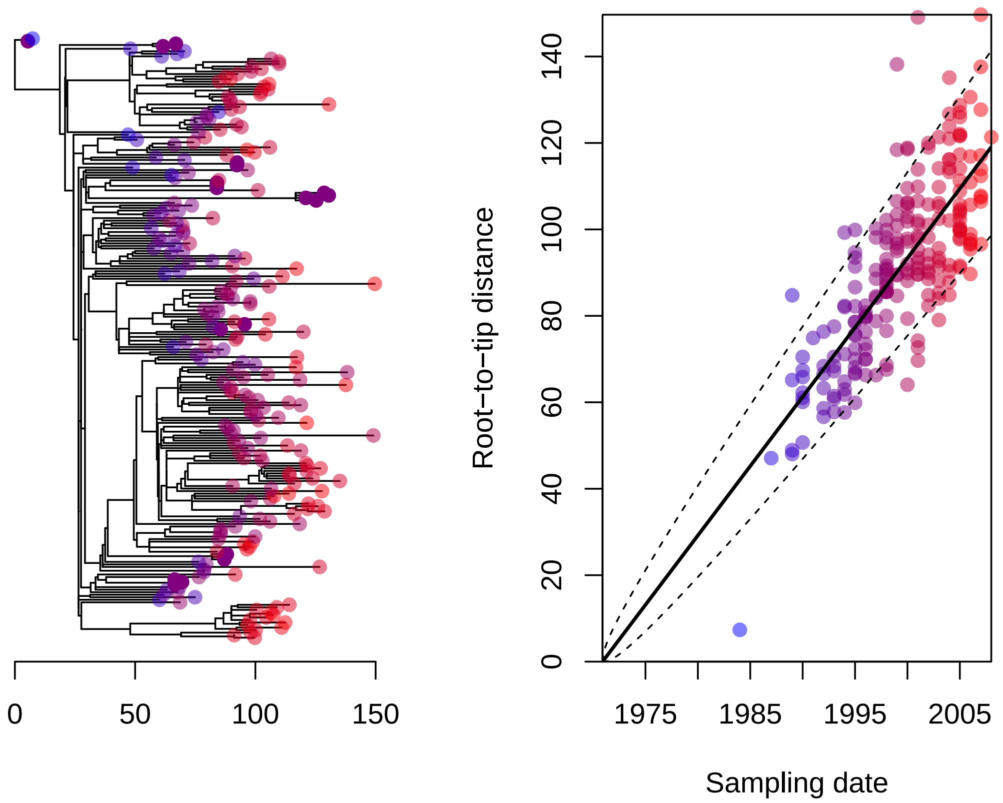

Figure S12. Linear regression analysis of root-to-tip distances versus isolation dates, based on the Gubbins tree of the *Streptococcus pneumoniae* PMEN1 dataset.
